# Inhibition of autophagy-lysosomal function exacerbates microglial and monocyte lipid metabolism reprograming and dysfunction after brain injury

**DOI:** 10.21203/rs.3.rs-7682363/v1

**Published:** 2025-10-21

**Authors:** Amir A Mehrabani-Tabari, Nivedita Hegdekar, Brian R Herb, Sazia Arefin Kachi, Chinmoy Sarkar, Sagarina Thapa, Dexter PH Nguyen, Yulemni Morel, Mehari M Weldemariam, Ludovic Muller, W Temple Andrews, Marcia Cortes-Gutierrez, Xiaoxuan Fan, Natarajan Ayithan, Olivia Pettyjohn-Robin, Sabrina Bustos, Lacey K Greer, Jessica T Gore, Maureen A Kane, Seth A Ament, Jace W Jones, Marta M Lipinski

**Affiliations:** 1 Department of Anesthesiology and Shock, Trauma and Anesthesiology Research Center, University of Maryland School of Medicine, Baltimore, MD, USA; 2 Department of Pharmaceutical Sciences, University of Maryland School of Pharmacy, Baltimore, MD, USA; 3 Institute for Genome Sciences, University of Maryland, School of Medicine, Baltimore, MD, USA; 4 Department of Neurobiology, University of Maryland School of Medicine, Baltimore, MD, USA; 5 Department of Psychiatry, University of Maryland School of Medicine, Baltimore, MD, USA; 6 Department of Pharmacology and Physiology, University of Maryland School of Medicine, Baltimore, MD, USA; 7 UM-MIND Institute for Neuroscience Discovery, University of Maryland School of Medicine, Baltimore, MD, USA; 8 Department of Microbiology and Immunology, University of Maryland School of Medicine, Baltimore, MD, USA; 9 University of Maryland Marlene and Stewart Greenebaum Comprehensive Cancer Center

## Abstract

CNS has an overall higher level of lipids than all tissues except adipose and contains up to 25% of total body cholesterol. Recent data demonstrate a complex crosstalk between lipid metabolism and inflammation, suggesting potential contribution of the lipid-rich brain environment to neuroinflammation. While recent data support the importance of brain lipid environment to inflammatory changes observed in age related chronic neurodegenerative diseases, *in vivo* interactions between lipid environment, lipid metabolism and neuroinflammation in acute brain disease and injury remain poorly understood. Here we utilize a mouse model of traumatic brain injury (TBI) to demonstrate that acute neurotrauma leads to widespread lipid metabolism reprograming in all microglial and brain associated and infiltrating monocyte populations. Additionally, we identify unique microglial and monocyte populations with higher degree of lipid metabolism reprograming and pronounced accumulation of neutral storage lipids, including cholesteryl esters and triglycerides. These lipids accumulate not only in lipid droplets but also in the microglial and monocyte lysosomes and are associated with lysosomal dysfunction and inhibition of autophagy after TBI. Our data indicate that lipid accumulation in these cells is the result of altered lipid handling rather than lipid synthesis and is triggered by phagocytosis of lipid-rich myelin debris generated after TBI. Finally, we use mice with autophagy defects in microglia and monocytes to demonstrate that further inhibition of autophagy leads to more pronounced lipid metabolism reprograming and exacerbated cellular lipid accumulation. Our data suggest a pathological feedback loop, where lipid phagocytosis causes inhibition of autophagy-lysosomal function, which in turn exacerbates cellular lipid retention, reprograming and inflammation.

## Introduction

Recent studies have revealed a complex bi-directional crosstalk between lipid metabolism and inflammation. This includes reports of immune stimuli altering cellular lipid composition and conversely, cellular lipid storage and lipid droplet formation promoting inflammatory responses.^1,2^ Deciphering the mechanisms of these interactions will be particularly important for understanding how lipid environment affects physiological and pathological immune responses in tissues high in lipid, such as the adipose, atherosclerotic plaques and the central nervous system (CNS), including brain and spinal cord.

Because of prevalence of branched cells with high membrane-to-volume ratios and presence of cholesterol-rich myelin, CNS has overall higher level of lipids than all tissues except the adipose and contains up to 25% of total body cholesterol.^3,4^ Altered expression of lipid metabolism genes is commonly observed in many age- and disease-associated microglial populations.^5–7^ Lipid metabolism reprograming is particularly pronounced in lipid droplet associated microglia (LDAM) which accumulate in the aged and Alzheimer’s disease (AD) brain.^1,8^ LDAM are highly pathogenic, with properties including inhibition of phagocytosis and high levels of inflammation. ^1^ In the context of AD accumulation of microglial lipid droplets are triggered by exposure to amyloid b and exacerbated by the presence of the AD-associated *APOE4* allele.^9,10^ It is less clear how their formation is initiated in the absence of amyloid pathology. It is also not known whether microglial lipid metabolism reprograming and/or LDAM generation are part of acute neuroinflammatory responses following infection or injury.

Traumatic brain injury (TBI) is a leading cause of death and disability worldwide, and a significant predisposing factor to development of neurodegenerative diseases later in life.^11^ An important component of TBI is exacerbated and prolonged inflammation. It involves several cell types, including both microglia and peripheral macrophages/monocytes infiltrating the brain after injury-induced blood-brain-barrier compromise.^12^ The reasons for the disproportionately high pro-inflammatory response after TBI remain poorly understood. We recently demonstrated that autophagy, a lysosome-dependent catabolic pathway necessary for degradation and recycling of proteins, protein aggregates, organelles and other cellular components,^13–15^ is inhibited after TBI and that multiple cell types including activated microglia and infiltrating monocytes are affected.^16–18^ In addition to its well-documented role in proteostasis and organelle quality control, autophagy has been shown to regulate inflammatory responses, with high levels of autophagy generally associated with anti-inflammatory, and low levels with pro-inflammatory phenotypes.^19,20^ Consistently, our data demonstrated that inhibition of microglial and monocyte autophagy contributes to excessive and prolonged neuroinflammation after TBI.^16^ Since recent data indicate that autophagy also participates in lipid catabolism by targeting and degrading lipid droplets through lipophagy,^21,22^ its inhibition in immune cells suggests potential interaction between autophagy, inflammation and lipid metabolism in the TBI brain.

The mechanisms behind autophagy inhibition in microglia and monocytes after TBI remain unknown. As the resident phagocytic cells of the brain, microglia are responsible for clearing dead cells and cellular debris including lipids, which are all delivered to lysosomes for degradation.^23,24^ In peripheral phagocytic cells such as atherosclerotic plaque macrophages, phagocytosis of certain lipids, in particular cholesterol, can cause lysosomal dysfunction.^25^ Similarly, myelin phagocytosis contributes to lysosomal dysfunction in multiple sclerosis (MS). In both atherosclerosis and MS lipid phagocytosis is also associated with accumulation of storage lipids and inflammation.^26–28^ Since TBI leads to generation of abundant myelin debris we hypothesized that similar mechanism could contribute to both the inhibition of autophagy and lipid metabolism reprograming after TBI.

We used multi-omics approach to investigate interaction between lipid metabolism, autophagy-lysosomal function and inflammation in monocytes after TBI. Our lipidomic data demonstrated that neutral lipids like triglycerides and cholesteryl esters accumulated in the activated microglial and monocytes in the vicinity of TBI lesion. We used single cell RNA sequencing (scRNA-seq) to demonstrated that TBI leads to widespread microglial and monocyte lipid metabolism reprogramming, which preferentially affected lipid handling as opposed to lipid synthesis genes. Additionally, our data identified microglial and monocyte populations with more pronounced lipid metabolism reprograming and demonstrated that they correspond to the lipid accumulating cells observed in the TBI lesion. Lipid accumulation in these cells was triggered by phagocytosis of lipid-rich myelin debris generated after TBI and occured not only in lipid droplets but also in lysosomes, leading to their dysfunction and inhibition of autophagy. Confirming interaction between autophagy, lipid metabolism and inflammation, lipid metabolism reprograming and cellular lipid accumulation after TBI were exacerbated in mice with autophagy defects in microglia and monocytes. Our data suggest that TBI triggers a pathological feedback loop, where lipid phagocytosis causes inhibition of autophagy-lysosomal function, which in turn exacerbates cellular lipid retention, reprograming and inflammation.

## Results

### TBI leads to accumulation of neutral lipids in microglia and infiltrating macrophages

**Figure 1.**
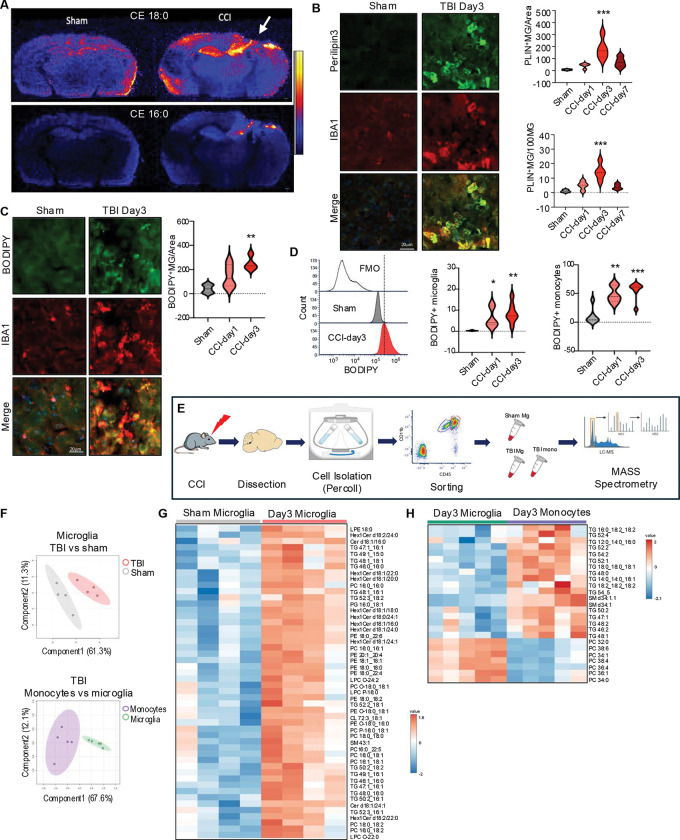
TBI leads to accumulation of neutral lipids in microglia and infiltrating macrophages. (A) Desorption Electrospray Ionization- Mass Spectrometry Imaging (DESI-MSI) of coronal mouse brain slices from sham and TBI (CCI model, day3), showing spatial distribution of cholesteryl esters 18:0 (cholesteryl stearate) and 16:0 (cholesteryl palmitate). (B) Immunostaining and quantification including time course of microglia/macrophages (IBA1, red) positive for lipid droplets protein, perilipin3 (green) in cortical sections from sham vs TBI mice (20X). Values are means ± SEM, n = 4 mice/group; *** P < 0.001 vs sham (one-way ANOVA with Tukey’s multiple comparisons test). (C) Immunostaining and quantification of neutral lipid (BODIPY, green) in microglia/macrophages (IBA1, red) in cortical sections from sham vs TBI. n = 4–5 mice/group; ** P < 0.01 vs sham (one-way ANOVA with Tukey’s). (D) Quantification of microglia and infiltrating macrophages accumulating neutral lipids (BODIPY) after TBI (day3). Left histogram demonstrating BODIPY signal shift from negative control (fluorescence minus one, FMO), in sham, and TBI microglia. Center & right quantification of BODIPY+ microglia and monocytes. n = 6–7 mice/group; * p>0.05, ** P<0.01, *** P<0.001 vs sham (one-way ANOVA with Tukey’s). (E) Experimental workflow for assessment of lipid composition of FACS isolated microglia and infiltrating monocytes from sham and TBI ipsilateral cortex. (F) Principal Component Analysis (PCA) scores plots demonstrating significant differences between lipid profiles of TBI vs sham microglia (top) and TBI monocytes vs TBI microglia (bottom) based on LC-MS/MS analyses. Each point represents sample form an individual mouse; 90% confidence intervals are shaded; n=4–5/group. (G-H) Heatmaps identifying differentially abundant lipids in TBI vs microglia (G) and TBI monocytes vs TBI microglia (H).

We used desorption electro-spray ionization - mass spectrometry imaging (DESI-MSI) to assess overall extent of TBI-induced changes in brain lipid content and distribution. Wild type C57BL/6 12-week-old male mice were subjected to moderate controlled cortical impact (CCI), a well-characterized mouse model of TBI. Brains were harvested 3 days after injury or sham surgery, a time point corresponding to peak inflammation and monocytes infiltration in the CCI model.^29^ We observed significant changes in brain lipid distribution after TBI (Figure S1A-F). This included prominent accumulation of neutral lipids such as cholesteryl esters and triglycerides, which was most pronounced in the vicinity of the TBI lesion (Figure 1A and Figure S1C). Accumulation of neutral lipids in the TBI tissue suggested increase in the formation of lipid storage organelles, the lipid droplets.^30^ To check whether mononuclear phagocytic cells including microglia and infiltrating macrophages accumulate lipid droplets after TBI, we immunostained brain sections with antibodies against lipid droplet coating protein perilipin-3 (PLIN3). We observed lipid droplet formation in IBA1^+^ monocytes starting on day1, with peak at day 3 and decline by day 7 after TBI (Figure 1B). Neutral lipid accumulation in monocytes was confirmed by staining with neutral lipid dye, BODIPY (Figure 1C). We used flow cytometry to determine whether the affected cells were resident brain microglia (CD11B^+^ CD45^int^) or infiltrating monocytes (CD11B^+^ CD45^hi^) (Figure S1G). Consistent with immunostaining results, we observed significantly higher number of cells accumulating BODIPY starting on day 1 and peaking on day 3 after TBI. While neutral lipid accumulation was observed in both resident microglia and infiltrating monocytes, it was significantly more pronounced in the infiltrating monocytes (CD45^hi^) populations (Figure 1D).

Our data suggested that following TBI mononuclear phagocytes including microglia and infiltrating macrophages accumulate neutral storage lipids such as triglycerides and cholesteryl esters. In cells other than adipocytes high level of storage lipid accumulation indicate overall lipid metabolism imbalance.^31^ To gain clearer understanding of the lipid signatures of these cells we used fluorescent activated cell sorting (FACS) to isolate microglia (CD11b^+^ CD45^int^) and infiltrating monocytes (CD11b^+^ CD45^hi^) from mouse perilesional cortex tissue (Figure 1E). Extracted lipids were analyzed using a targeted lipidomics method consisting of ultra-performance liquid chromatography (UPLC) coupled to selected reaction monitoring (SRM) on a tandem quadrupole mass spectrometer.^32^ We detected significant differences in lipid composition between microglia isolated from sham versus TBI mice as well as between microglia and infiltrating monocytes within the TBI group (Figure 1F,). Consistent with immunostaining and flow cytometry results, the most prominent group of lipids with increased abundance in microglia after TBI were triglycerides, a storage lipid species highly abundant in lipid droplets (Figure 1G, Table_S1). Triglyceride abundance was even higher in monocytes infiltrating the brain tissue after TBI (Figure 1H, Table_S2). Other lipid species with increased abundance in TBI microglia and monocytes included hexosylceramides and sphingomyelins, lipids highly enriched in myelin,^33^ suggesting that internalized myelin debris from the TBI lesion could be a contributing source of accumulated lipid.

### TBI causes pronounced lipid metabolism reprogramming in microglia and macrophages

**Figure 2.**
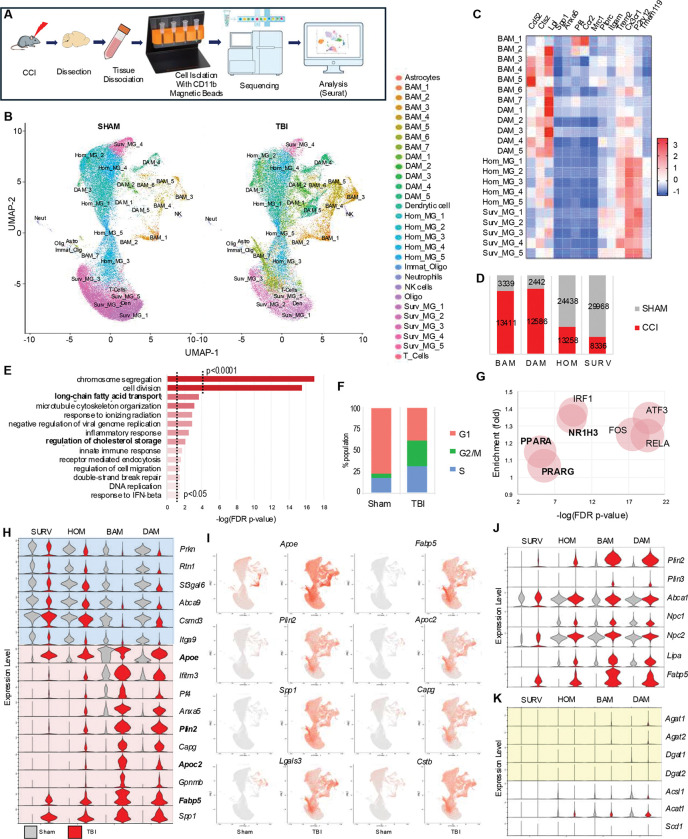
TBI causes pronounced reprogramming of lipid metabolism in microglia and macrophages. (A). Experimental workflow for assessment of transcriptomic changes in isolated CD11B^+^ cells from from sham vs TBI (day3) mouse brain. (B) Uniform manifold approximation (UMAP) demonstrating identification of 29 transcriptionally distinct clusters and their manually assigned identities. (C) Heatmap representing expression levels of canonical homeostatic and disease associated markers used for cluster identity assignment. Color coding is based on z-score scaling. (D) Bar chart showing distribution of the four main cell populations: surveillant microglia (SURV), homeostatic microglia (HOM), disease associated microglia (DAM), and brain associated macrophages (BAM) in sham vs TBI brain. (E) Gene Ontology Biological Process (GO:BP) enrichment analysis for genes differentially expressed in TBI vs sham. (F) Seurat Cell Cycle analysis demonstrating higher proportion of cells in S and G2/M phase of the cell cycle after TBI vs majority of cells in G1 phase in sham. (G) Bubble plot representing transcription factors link (TFlink) analysis. Identified as upstream regulators of DEGs in TBI vs sham. The size of each bubble is proportionate to the number of DEGs controlled by the transcription factor. (H) Violin plots demonstrating expression of top DEGs downregulated (blue) and upregulated (pink) in TBI in the four main microglial and macrophage cell populations. Highlighted in bold are lipid handling genes. (I) Feature plots demonstrating wide-spread changes in lipid metabolism (*Apoe*, *Fabp5*, *Plin2*, and *Apoc2*), microglial activation (*Spp1*), phagocytosis (*Capg*), and lysosomal fuction (*Lgals3* and *Cstb*) after TBI. (J-K) Violin plots demonstrating expression of lipid handling (J) and lipid synthesis (K) genes in the four main microglial and macrophage cell populations. Genes involved in triglyceride synthesis are highlighted in yellow in (K).

We performed single cell RNA sequencing (scRNA-seq) to gain a higher-resolution picture of the changes in microglia and monocytes during the acute phase of TBI. We focused on day 3 after TBI, a period of rapid monocyte cell proliferation and inflammatory reprogramming.^34^ CD11B^+^ cells were isolated with magnetic beads from the ipsilateral hemispheres of TBI vs sham mice (4/group) for 10x Genomics scRNA-seq, yielding 111,458 cellular transcriptomes after initial quality control (Figure 2A and Figure S2A). Cell clustering with Seurat revealed 29 transcriptionally distinct populations, which were annotated based on established marker genes and our experience with inflammatory changes in the CCI model^1,16,35–38^ (Figure 2B–C and Figure S2B). 22 out of 29 clusters (107,778 out of 111,458 sequenced cells, 96.7% enrichment) showed strong microglial/monocyte signatures expected for CD11B cells including 5 homeostatic microglia (Hom_MG_1–5), 5 surveillance microglia (Surv_MG_1–5), 5 disease associated microglia (DAM_1–5), and 7 brain associated macrophages (BAM_1–7). The remaining 7 minor clusters (each under 0.5% of total cell population) were excluded from further analysis.

As expected, the majority of cells from sham mice belonged to homeostatic and surveillance microglial populations, with minor contribution of BAM and even fewer DAM. TBI lead to strong enrichment of the DAM and BAM populations, accompanied by a proportional decrease in homeostatic/surveillance microglia (Figure 2D). For initial analyses we compared 18318 differentially expressed genes (DEGs) in TBI and sham animals across all microglia/monocyte populations (Table_S3). Gene Ontology Biological Process (GO:BP) enrichment analysis demonstrated overrepresentation of DEGs related to cell proliferation, which was confirmed by Seurat cell cycle analysis (Figure 2E–F, Figure S2C, Table_S4). Other overrepresented categories included inflammatory responses, especially innate immunity known to be strongly activated after TBI, as well as terms related to lipid metabolism, especially fatty acid and cholesterol processing (Figure 2E). This was confirmed by transcription factor (TFlink) analyses, which in addition to canonical regulators of cell proliferation (FOS, ATF3) and inflammatory responses (RELA, IRF1) identified lipid metabolism regulators such as LXR (NRF1H3) and PPAR (PPARA, PPARG) as upstream drivers of the observed transcriptional changes (Figure 2G). These data suggest that TBI leads to general reprograming of microglial/monocyte lipid metabolism. To exclude the possibility that these results were driven by strong changes in a specific cell population, we compared expression of top up- and down-regulated genes in homeostatic and surveillance microglia, DAMs and BAMs from TBI versus sham mice. The top ten genes upregulated after TBI included four genes involved in lipid homeostasis (*Apoe*, *Fabp5*, *Plin2*, *Apoc2*), which were increased in most cell populations, with BAM and DAM affected to the highest extent, and surveillance microglia the least (Figure 2H–I, Figure S3A). Other genes dysregulated in multiple populations included those involved in phagocytosis (*Spp1*, *Capg*), lysosomal function (*Ctsb*) and lysosomal damage (*Lgals3*). Consistent with overall increase in DAM and BAM in TBI, many of these top markers are known DAM genes.

TLR4 signaling, which is strongly activated after TBI,^39^ has been shown to activate lipid synthesis leading to neutral lipid accumulation in TLR4 agonist treated mouse bone marrow derived macrophages (BMDM).^2^ To determine if this TLR4-dependent activation of lipid synthesis may be responsible for the observed accumulation of neutral lipid after TBI, we compared expression of genes involved in different stages of lipid metabolism. Unexpectedly, while lipid handling genes were strongly upregulated in all cell types (Figure 2J), expression of lipid synthesis genes, including those involved in triglyceride synthesis, was affected to a much lesser degree (Figure 2K, Figure S3B). These data suggest that TBI-induced microglial and monocyte neutral lipid accumulation may be caused by altered cellular lipid handling rather than increased lipid synthesis. Finally, while our data identified transcription factors involved in regulation of lipid metabolism as some of the top upstream regulators responsible for the TBI-induced transcriptional changes, expression levels of mRNAs encoding these factors was not altered (Figure S3C).

### Lipid reprogramming is more pronounced in lipid-accumulating microglia and macrophages

**Figure 3.**
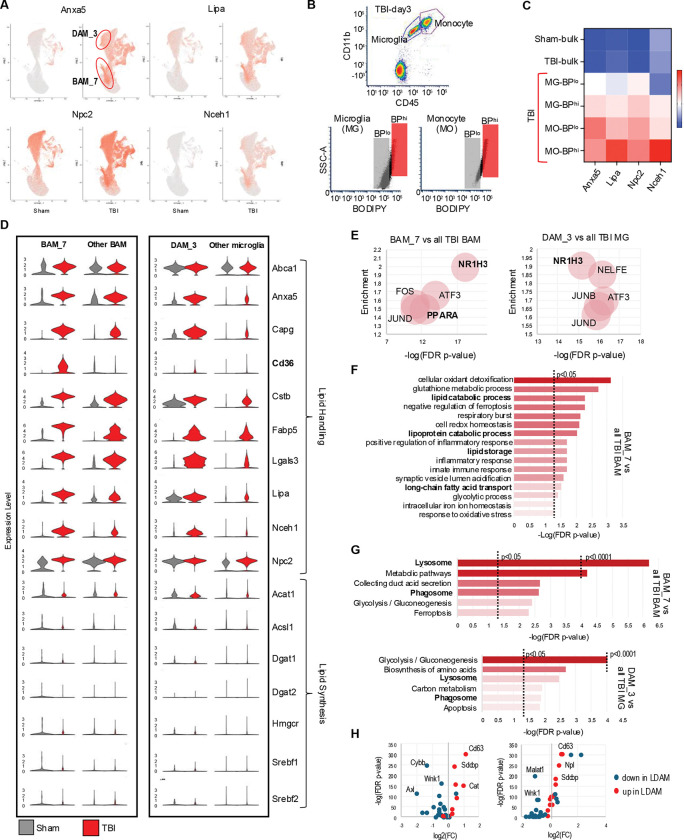
Lipid reprogramming is more pronounced in lipid-accumulating microglia and macrophages. (A) Feature plots for genes involved in lipid storage and processing (*Lipa*, *Npc2*, and *Nceh1*) and phagocytosis (Anxa5) identifying BAM_7 and DAM_3 as clusters with more pronounced lipid metabolism reporogramming. (B) Flow cytometry gating strategy for isolation of microglia (CD11B^+^CD45^int^) and monocytes (CD11B^+^CD45^hi^) with either low (BP^lo^) or high (BP^hi^) lipid load used for RNA isolation. (C) Heatmap of RTqPCR analysis of TBI-induced expression changes in DAM_3/BAM_7 markers from panel A in microglial (MG) and monocytes (MO) cells isolated using strategy from panel B. Increased expression levels in BPhi microglia and monocytes as compared to corresponding BPlo cells, indicated that that they represent DAM_3 and BAM_7 populations, respectively. (D) Violin plots representing expression levels of lipid handling genes (top) and lipid synthesis genes (bottom) in BAM_7 vs all other BAMs (left) and in DAM_3 vs all other microglia (right). (E) TFlink analysis identifying top five transcription factors responsible for DEGs in BAM_7 vs all other BAMs (left) and DAM_3 vs all other microglia (right). The size of each bubble is proportionate to the number of DEGs genes controlled by the transcription factor. (F) Gene Ontology Biological Process (GO:BP) enrichment analysis for genes differentially expressed in BAM_7 vs all other BAMs following exposure to TBI. Lipid metabolism terms are highlighted in bold. (G) KEGG pathway analyses of BAM_7 vs all other TBI BAMs and DAM_3 vs all other TBI microglia. Terms related to lysosomal function are highlighted in bold. (H) Volcano plots demonstrating altered expression of LDAM genes^1^ in BAM_7 vs all other TBI BAMs and DAM_3 vs all other TBI microglia.

While we observed TBI-induced lipid metabolism reprograming in all microglial and monocyte populations, some clusters were affected to a larger extent. In particular, BAM cluster 7 (BAM_7) and DAM cluster 3 (DAM_3) showed more pronounced upregulation of lipid handling genes as compared to other TBI cell populations (Figure 3A, Figure S3A, Table_S5). To determine whether these clusters may correspond to the lipid accumulating cells we observed after TBI (Figure 1), we used FACS to isolate microglial and monocyte population with high versus low levels of lipid accumulation (BODIPY^high^ and BODIPY^low^, respectively) from ipsilateral TBI day 3 cortices (Figure 3B). BODIPY^high^ microglia and monocytes showed elevated expression of BAM_7 and DAM_3 markers as compared to corresponding BODIPY^low^ cells (Figure 3C). Consistent with very low numbers of BODIPY positive cells in sham animals, DAM_3 and BAM_7 clusters were almost exclusively present in TBI samples. These data indicate that BAM_7 and DAM_3 represent the lipid-accumulating microglia and monocytes in the TBI brain.

To better understand the properties of lipid accumulating monocytes and microglia, we analyzed BAM_7 and DAM_3 clusters as compared to, respectively, all other BAMs and all other microglial cell populations. Both clusters demonstrated exacerbated induction of genes involved in lipid storage and handling and in lysosomal function after TBI (Figure 3D). Notable were high expression levels of neutral cholesteryl ester hydrolase 1 (*Nceh1*), the enzyme responsible for converting neutral cholesterol esters to free cholesterol. We speculate that this could be a compensatory response to cholesteryl ester accumulation we observed in our DESI-MSI analyses (Figure 1A). We also noted almost exclusive upregulation of the scavenger receptor *Cd36* in BAM_7 after TBI which aligns with a recent report showing that *Cd36* positive macrophages in mouse model of ischemic stroke exhibited enhanced expression of phagocytic and lipid-handling genes.^40^ Taken together, these data suggest that high expression of *Cd36* may be a marker of lipid-loaded monocyte population after TBI.

Pathway analyses confirmed more pronounced dysregulation of lipid metabolism pathways in BAM_7 and DAM_3 as compared to other TBI BAM and microglial populations. This included identification of lipid metabolism transcription factors among the top 5 upstream regulators responsible for the observed transcriptional changes in both clusters (Figure 3E), and enrichment in processes related to cellular lipid catabolism, transport and storage in GO:BP analysis of BAM_7 (Figure 3F). These data are consistent with changes in lipid processing rather than synthesis as drivers of lipid accumulation after TBI. Other overrepresented processes included those involved in redox homeostasis, mitochondrial function and iron homeostasis, all previously reported to be affected after TBI. KEGG pathway analyses of both BAM_7 and DAM_3 included lysosomal and phagocytic pathways among the top six most significantly overrepresented, suggesting that in addition to changes in lipid metabolism, cells in both clusters may have altered lysosomal function as compared to other TBI BAM and microglial populations (Figure 3G). Lipid droplet associated microglia (LDAM) accumulate and play a pathological role during brain during aging and in neurodegenerative diseases such as AD.^1^ We compared expression of genes reported as most significantly up- and down-regulated in LDAM to those altered in BAM_7 and DAM_3. We observed overall strong correlation, with over 60% of LDAM genes significantly altered in both BAM_7 and DAM_3 (Figure 3H), suggesting that lipid accumulating microglia and monocytes acutely forming after injury share many features with those present in the aged and AD brain.

Recent data demonstrate that interferon signaling is an important contributor to TBI neuroinflammation.^34,41^ Consistently, we identified interferon regulatory factor IRF1 as one of the top upstream transcriptional regulators in TBI (Figure 2G). To investigate the relationship between lipid metabolism and interferon responses, we assessed expression of interferon response genes among identified cell populations. Our data demonstrate that unlike lipid metabolism, upregulation of interferon genes is restricted to specific BAM and DAM clusters (Figure S3D-E). Overall, BAM populations displayed more pronounced interferon responses as compared to DAM. However, we identified DAM_4 as displaying pattern of interferon gene expression similar to BAM; and DAM_2, which upregulated a unique set of interferon response genes, including *Ifit2* and *Ifit3*. Neither BAM_7 nor DAM_3 showed strong interferon responses as compared to other BAM and microglia, respectively. This suggests that lipid accumulation and upregulation of interferon pathways occur in different cell populations.

### Lipid accumulates in the lysosomes of microglia and macrophages after TBI

**Figure 4.**
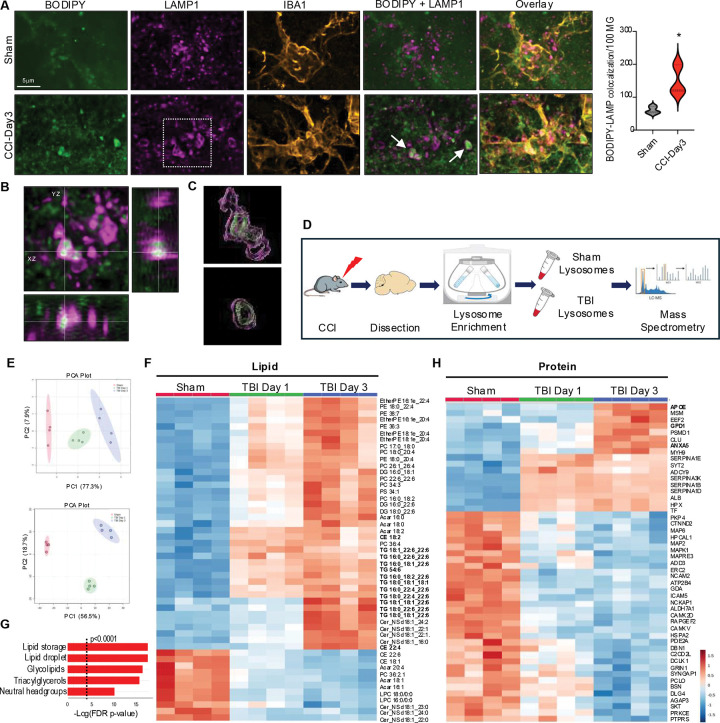
Neutral lipids accumulate in the lysosomes of microglia and macrophages after TBI. (A) Confocal images and quantification demonstrating accumulation of neutral lipid (BODIPY, green) in lysosomes (LAMP1, magenta) of microglia/macrophage cell (IBA1, orange) in coronal brain sections from TBI vs sham mice (100X); P < 0.05 using Student’s t-test. (B) Orthogonal views of indicated image area from panel A demonstrating localization of BODIPY within LAMP1 lysosomes. (C) 3D reconstruction of BODIPY engulfment inside lysosomes indicated with white arrow in panel A. (D) Experimental workflow for assessment of lipid and protein composition of lysosomal fractions from peri-lesion cortical tissue of TBI vs sham. (E) PCA plots demonstrating significant differences in lipid (top) and protein (bottom) composition between lysosomal fractions from sham vs TBI-day3 cortices based on LC-MS/MS analyses. Each point represents sample from an individual mouse; 90% confidence intervals are shaded; n = 4/group. (F) Heatmap identifying lipids differentially abundant in lysosomal fractions from TBI-day1 and TBI-day3 vs sham cortex based on ANOVA. (G) Lipid ontology (LION) enrichment analysis of differentially abundant lipids in TBI-day3 vs sham. (H) Metaboanalyst generated heatmap identifying proteins differentially abundant in lysosomal fractions from TBI-day1 and TBI-day3 vs sham cortex based on ANOVA.

Our analyses of BAM_7 and DAM_3 indicated that both are characterized by changes in phagocytosis and lysosomal function. We investigated spatial relationship between lipid accumulation and lysosomes by immunofluorescence for neutral lipid (BODIPY) and lysosomal membrane marker LAMP1 in IBA1^+^ microglial and monocyte cells (Figure 4A–B). We observed significant lysosomal accumulation of neutral lipid in phagocytes after TBI, which was further verified by 3D reconstruction of lipid-containing lysosomes (Figure 4C, Figure S4A). These data demonstrate that, in addition to lipid droplets, neutral lipids accumulate in the lysosomes rather than being processed for export to other cellular compartments, suggesting lysosomal dysfunction. To determine how TBI affects lysosomal composition and function, we isolated lysosome enriched fractions from perilesional cortical tissue at 1 and 3 days after TBI or sham surgery^17^ for LC-MS/MS lipidomic and proteomic analyses (Figure 4D). We detected significant differences in both lysosomal lipid and protein composition after TBI (Figure 4E). Lipidomic results demonstrated lysosomal accumulation of neutral lipids, including triglycerides and cholesteryl ester species (Figure 4F, Figure S4B-C, Table_S6). Significant enrichment for storage lipids normally observed in lipid droplets was confirmed by LION analysis (Figure 4G). Additionally, we observed increased abundance of ceramides, which together with cholesteryl esters suggested myelin as the source of lipids accumulating in TBI lysosomes (Figure 4F). Consistent with its pronounced transcriptional upregulation and its known role as the major lipid carrier in the brain,^42^ our proteomic data identified APOE as the top protein enriched in day 3 TBI lysosomes (Figure 4H, Table_S7). Other increased proteins include ANXA5, a phagocytic marker transcriptionally elevated after TBI, and glycerol-3-phosphate dehydrogenase 1 (GPD1), an enzyme involved in triglyceride metabolism and the cause of transient infantile hypertriglyceridemia.^43^ Finally, we observed higher abundance of several members of the serpin family protease inhibitors (SERPINA1B, 1D, 1E, and 3K). Serpins irreversibly inhibit extracellular serine proteases involved in coagulation, cell migration and inflammatory responses^44^ and they entrapment in the lysosomes could contribute to TBI neuroinflammation.

### Myelin phagocytosis inhibits lysosomal function and autophagy after TBI

**Figure 5.**
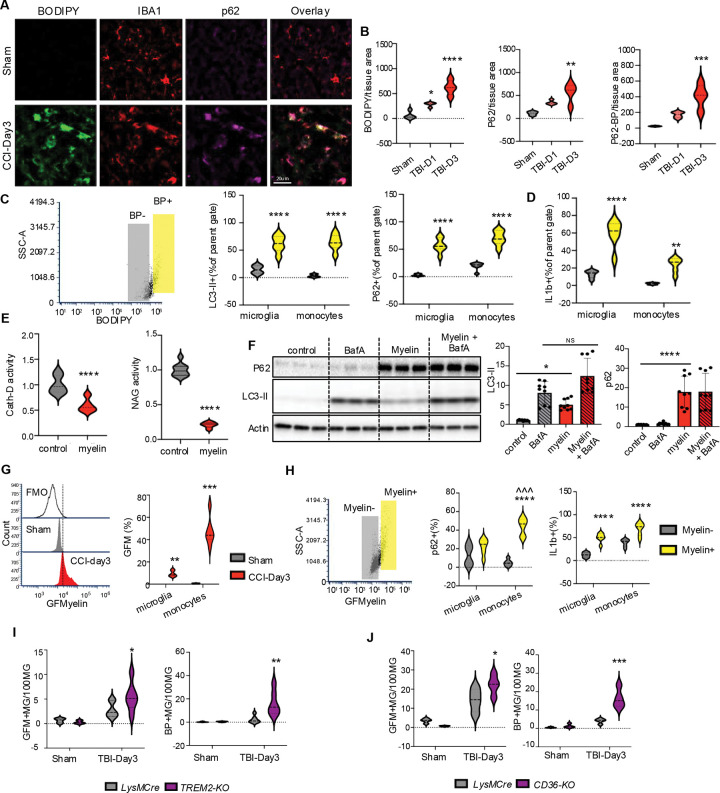
Myelin phagocytosis leads to inhibition of lysosomal function and autophagy after TBI. (A) Immunostaining demonstrating colocalization between lipid accumulation (BODIPY, green) and inhibition of autophagy (p62, magenta) in microglia/macrophages (IBA1, red) in cortical sections from TBI vs sham cortex (20X). (B) Quantification of data from panel A. n = 5 mice/group; * P < 0.05, ** P < 0.01, *** P < 0.001, **** P < 0.0001 (one-way ANOVA with Tukey’s). (C) Flow cytometry scatter plot representing gating strategy dividing TBI monocytes into BODIPY ^hi^ and BODIPY ^lo^ sub-populations. (D) Flow cytometry-based comparisons of autophagy (LC3 and p62, left and middle) and inflammatory (IL1β, right) markers in TBI microglia and monocytes with high (BODIPY^+^) vs low (BODIPY^−^) lipid accumulation. n = 6 mice/group; * P < 0.05. ** P < 0.001, **** P < 0.0001 (two-way ANOVA with Tukey’s). (E) Quantification of activity for lysosomal enzymes Cathepsin-D and NAGLU in BMDMs treated with purified mouse myelin (100 μg/ml – 24 hours) vs control. Values are mean ± SEM, n = 9 (3 replicates of 3 independent experiment); **** P < 0.0001 (Student’s t-test). (F) Western blots and quantification of autophagy markers p62 and LC3-II in BMDM treated with myelin vs control. Where indicated, Bafilomycin A (50 nM) was added for last 4 hours. Bar graphs are mean ± SEM, n = 9 (3 replicates of 3 independent experiments); * P < 0.05, **** P < 0.0001 (two-way ANOVA with Tukey’s). (G) Flow cytometry-based quantification of myelin (GreenFluoroMyelin, GFMyelin) accumulation in microglia and monocytes. Left - histogram demonstrating GFM signal shift from FMO in sham and TBI microglia. Right – quantification of GFM^+^ microglia and monocytes in TBI vs sham. n = 6 mice/group; * P < 0.05. ** P < 0.001, ***P<0.001 vs sham (Student’s t-test). (H) Flow cytometry scatter plot demonstrating identification of myelin^+^ and myelin^-^ monocyte sub-populations (left) and comparisons of autophagy marker p62 (center) and inflammation marker IL1β (right) in TBI microglia and monocytes with high vs low myelin accumulation. n = 6 mice/group. (I-J) Flow cytometry comparisons of myelin (GFMyelin, left plots) and neutral lipid (BODIPY, right plots) accumulation in mice deficient for *Trem2* (I) or conditionally deficient for *Cd36*. (J) vs corresponding WT controls. n = 6 mice/group. (two-way ANOVA with Tukey’s).

**Figure 6.**
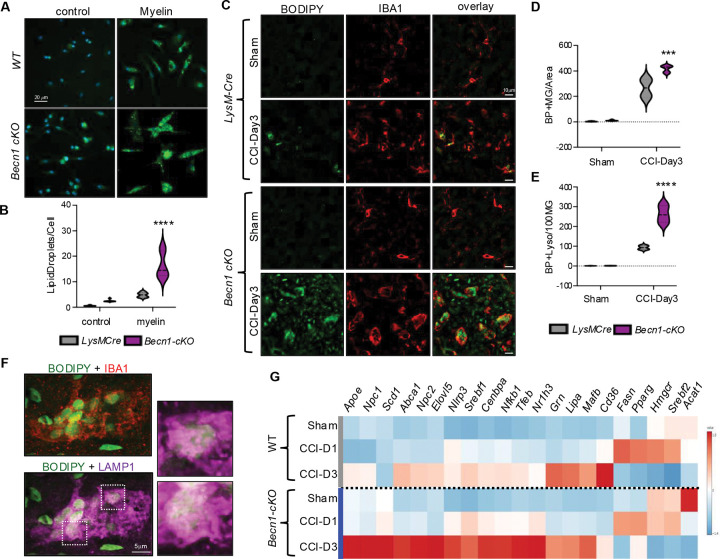
Autophagy deficiency exacerbates lipid accumulation and lipid metabolism reprograming. (A) BODIPY staining imaging of BMDMs (20x, scale bar 25 μm) from *LysMCre* (wild type) or *Beclin1-cKO* mice, following myelin treatment (100 μg/ml, 24 hours). (B) Quantification of data from panel A. n = 6 (3 replicates of 2 independent experiments); **** P < 0.0001 vs *LysMCre* (two-way ANOVA with Tukey’s). (C) Immunostaining demonstrating increased lipid (BODIPY, green) accumulation in microglia/macrophages (IBA1, red) in cortical sections of *Becn1-cKO* mice exposed to TBI as compared to *LysMCre* (wild type) mice (20X). (D-E) Quantification of IBA^+^ cell also positive for BODIPY per 100 IBA+ cells (20X images quantified in D) and colocalization of BODIPY and LAMP1 (60X images quantified in E). n = 4 mice/group; ** P < 0.01 vs *LysMCre* (two-way ANOVA with Tukey’s). (F) Confocal images demonstrating accumulation of neutral lipids (BODIPY, green) in lysosomes (LAMP1, magenta) of microglia (IBA1, red) in *Becn1-cKO* TBI brain sections. (G) RT-qPCR based heatmap comparing gene expression changes in lipid markers in peri-lesion cortical tissues from *LysMCre* (wild type) and *Beclin1-cKO* sham, TBI-day1, and TBI-day3 mice; n = 6 mice/group.

We recently demonstrated that autophagy is inhibited in both resident microglia and infiltrating monocytes after TBI, resulting in exacerbation of inflammatory responses.^16^ Both the timing (peak at day 3 post injury) and cell type distribution (higher in infiltrating monocytes than in microglia) of autophagy inhibition correlated with that observed for lipid accumulation after TBI (Figure 1). Since correlation between lipid accumulation and inhibition of autophagy flux in macrophages has been observed in the context of atherosclerosis,^45^ we tested whether autophagy flux is also inhibited in lipid accumulating monocytes of TBI lesion. Immunofluorescence staining showed that ~72% of neutral lipid positive (BODIPY^+^) monocytes (IBA^+^) also accumulated high levels of autophagy adaptor p62, indicative of decreased autophagic cargo degradation (Figure 5A–B). Autophagy flux inhibition in lipid accumulating mononuclear phagocytes was verified using flow cytometry, where levels of both p62 and LC3 were significantly higher in BODIPY^+^ microglia and infiltrating monocytes in the TBI brain as compared to corresponding BODIPY^−^ cells (Figure 6C). Moreover, BODIPY^+^ populations had higher levels of proinflammatory cytokine IL1β (Figure 5D). This effect was more pronounced in infiltrating monocytes compared with microglia which is consistent with the relationship between autophagy deficiency and proinflammatory phenotype polarization after TBI.^16^

TBI causes generation of abundant myelin debris.^46^ We hypothesized that phagocytosis of fragmented myelin may contribute to lipid accumulating and lysosomal dysfunction after TBI. To confirm the effect of phagocytosed myelin on monocyte lysosomes, we isolated mouse bone marrow derived macrophages (BMDM)^47^ and exposed them to purified mouse myelin.^48^ Myelin treatment led to lysosomal accumulation of myelin, which was prevented by phagocytosis inhibitor Dynasore (Figure S5A-B). Additionally, we observed lipid droplet accumulation similar to that observed after TBI (Figure S5C). Consistent with the ability of phagocytosed myelin to inhibit lysosomal function, myelin treatment significantly decreased activity of lysosomal enzymes CathepsinD and N-acetyl-glucosaminidase in BMDM (Figure 6E). Furthermore, myelin led to inhibition of autophagy, as demonstrated by accumulation of p62, and by LC3-II flux assay (Figure 5F, Figure S5D). We used flow cytometry to demonstrate accumulation of myelin (FluoroMyelin) in both resident microglia and infiltrating monocytes after TBI (Figure 5G). Similar to neutral lipid accumulation, myelin accumulation was more pronounced in infiltrating macrophages as compared to resident microglia. Furthermore, within TBI microglial and monocyte cells with high levels of accumulated myelin (Myelin+) displayed higher extent of autophagy inhibition (p62) and higher inflammation (IL1β, Figure 5H). These data support the hypothesis that phagocytosis of myelin generated after TBI causes lysosomal lipid accumulation and inhibition of lysosomal function, which in turn leads to inhibition of autophagy.

Several receptors, including TREM2 and the scavenger receptor CD36, have been implicated in phagocytosis of myelin in different models.^23,49^ We used mice with global deficiency for *Trem2* (*Trem2*^−/−^) or conditional deficiency for *Cd36* in microglia and monocytes (*LysM-cre/Cd36-flox*) to determine their function in myelin phagocytosis and regulation of lipid accumulation after TBI. Surprisingly, both *Trem2* and *Cd36* deficient microglia and monocytes showed increase in myelin phagocytosis and neutral lipid accumulation after TBI (Figure 5I–J However, the inflammatory responses for the two genotypes where not the same, with TNFa levels significantly higher in *Cd36* but not *Trem2* knock out microglia and monocytes as compared to wild type. IL1β levels were not altered in either genotype (Figure S6A-B).

### Autophagy deficiency exacerbates lipid accumulation and lipid metabolism reprogramming

Our data indicate that accumulation of myelin-derived lipids in the lysosomes leads to inhibition of autophagy after TBI. Autophagy has been also shown to participate in lipid homeostasis through lipophagy, which targets lipid droplets for lysosomal degradation and promotes cellular lipid efflux over storage.^45,50,51^ We hypothesized that autophagy deficiency would exacerbate lipid accumulation after TBI, creating a pathological feedback loop. We tested this hypothesis *in vitro* by exposing BMDMs from *LysM-cre/Becn1-flox* (*Becn1-cKO*) and *LysM-Cre* control mice to purified myelin and assessing lipid accumulation by BODIPY staining. We observed more lipid accumulation in *Becn1-cKO* BMDMs as compared to control both in the absence and presence of myelin, suggesting that autophagy deficiency is sufficient to promote lipid accumulation (Figure 6A–B). To test this *in vivo*, we subjected *Becn1-cKO* and *LysM-cre* control mice to CCI, followed by assessment of lipid accumulation at 3 days after injury. We observed significantly higher number of BODIPY^+^ microglia and macrophages (IBA1^+^) in brain tissue of injured *Becn1-cKO* as compared to *LysM-cre* control mice (Figure 6C–D). Staining for lipid droplet protein Perlipin 3 (PLIN3) demonstrated exacerbated lipid droplet formation in autophagy deficient monocytes after TBI (Figure S6C). *Becn1-cKO* mice also showed more lipid accumulation within lysosomes (Figure 6E–F). Finally, real-time qPCR analyses of perilesional cortex demonstrated exacerbated upregulation of lipid handling genes in injured *Becn1-cKO* as compared to *LysM-cre* mice, suggesting exacerbated reprograming of lipid metabolism in autophagy deficient monocytes after TBI (Figure 6G).

## Discussion

Recent data indicate an important contribution of altered lipid metabolism to microglial dysfunction during brain aging and in age-related neurodegenerative diseases such as AD.^1,5–7,10,52^ Much less is understood about the interactions between brain lipid environment, lipid metabolism and neuroinflammation during acute response to injury. Our data demonstrate that TBI leads to neutral lipid accumulation and rapid reprograming of lipid metabolism in microglia, brain associated macrophages and monocytes infiltrating the brain after injury-induced BBB disruption.^12^ Unlike aging and AD, where lipid reprograming is restricted to specific microglial sub-populations,^35^ after TBI virtually all microglia and monocytes in the ipsilateral hemisphere are affected to at least some extent. This likely reflects the overall breadth and intensity of microglial and monocyte inflammatory response after acute injury. Our data also support the notion that inflammatory and lipid metabolism responses are intimately linked, both under chronic and acute conditions.

While TBI-induced lipid metabolism reprograming was most pronounced in BAM and DAM populations, our data also revealed that homeostatic microglia responded more strongly as compared to surveillance microglia.^35^ The latter were defined as microglia enriched in the uninjured brain and expressing homeostatic as well as some activation markers. It is not clear why these populations may have attenuated lipid metabolic responses to injury as compared to homeostatic microglia. One possibility is that higher baseline activity of surveillance microglia makes them less prone to exaggerated responses to additional stimuli. Another, that homeostatic versus surveillance populations may be differentially distributed in respect to the injury site.^16^ Additional studies, such as spatial transcriptomic analyses of the injured brain will be necessary to differentiate between these possibilities.

In addition to demonstrating wide-spread lipid metabolism reprogramming after TBI, we used multi-omics approach to identify specific microglial (DAM_3) and monocyte (BAM_7) populations with exacerbated lipid metabolism changes and characterized by accumulation of neutral storage lipids such as cholesteryl esters and triglycerides. Inflammatory signaling through Toll-like receptor (TLR) activation has been previously shown to affect the lipidome of macrophages *in vitro*, with TLR4 stimulation specifically leading to neutral lipid accumulation and upregulation of triglyceride synthesis.^2^ This suggests that the lipidomic remodeling observed postTBI may be partly a downstream consequence of TLR signaling triggered by damage-associated molecular patterns (DAMPs) released during brain injury. However, we also observed significant differences. Our analyses indicate that after TBI lipid handling pathways are affected to a greater extent as compared to lipid synthesis. This was apparent both in overall analyses of microglial/monocyte responses as well as in lipid accumulating populations such as BAM_7 and DAM_3. This suggests that defects in lipid handling and efflux rather than activation of lipid synthesis may be the predominant mechanism leading to microglial/monocyte lipid accumulation after TBI. The reasons for this differential reprograming are not clear. While it is possible that it could reflect differences between cell types, overall reprograming patterns in monocytes infiltrating the brain after TBI resembled those observed in microglia. Additionally, while TLR activation by DAMPs plays an important role in activation of interferon signaling pathways,^53,54^ our analyses indicate that microglial/monocyte populations accumulating lipid after TBI are distinct from those with most pronounced activation of interferon responses. An alternative possibility is that the lipid-rich environment of the brain parenchyma interacts with pro-inflammatory stimuli such as TLR ligands to modulate cellular responses. This is supported by our and other data demonstrating that myelin phagocytosis leads to cellular lipid accumulation and altered expression of lipid metabolism and inflammatory genes in monocytes and microglia.^25,55^ While further analyses will be necessary to decipher these interactions, together, our findings and those of Hsieh et al.^2^ point to lipidome remodeling as a conserved response mechanism in macrophage-lineage cells across different inflammatory contexts, reinforcing the role of lipid metabolism as both a marker and mediator of inflammation.

Our data indicate that after TBI monocytes show more pronounced lipid accumulation and lipid metabolism reprograming as compared to activated microglia. Infiltrating monocyte-derived macrophages show stronger phagocytic uptake of apoptotic neurons and myelin fragments, which are rich in phospholipids, sphingolipids and cholesterol. Once internalized, these lipids are processed in the lysosomes and eventually stored in lipid droplets, especially when catabolic or efflux pathways are overwhelmed or dysfunctional. Additional factor contributing to the more pronounced phenotype in the infiltrating cells may be that unlike microglia, they have not been exposed to the unique brain lipid milieu. Recent studies show that infiltrating macrophages in several models of CNS injury can exhibit a “foamy” phenotype marked by lipid accumulation, increased levels of lipid droplet-associated proteins like PLIN2 and SOAT1, and dysregulation of cholesterol efflux.^25,56,57^ The impaired lipid handling may also be further amplified by environmental cues in the TBI lesion such as oxidized lipids, which potentiate scavenger receptor activity and lipid droplet formation.^58^ Intriguingly, HIF-1 signaling was one of the top hits in DAM_3 pathway analysis, which is in agreement with a recent observation that lipoprotein-derived fatty acids generated through LIPA activity stabilize HIF-1a independent of hypoxia.^59^ Therefore, the enhanced lipid burden in infiltrating macrophages may reflect both higher phagocytic uptake and lower capacity for intracellular lipid metabolism in these recruited cells.

CD36 and TREM2 receptors have been both previously implicated in myelin phagocytosis^23,56^. The unexpected increase in both myelin and lipid accumulation observed in *Cd36* and *Trem2* deficient microglia and monocytes following brain trauma may reflect redundancy and compensatory mechanisms among scavenger receptors. Additionally, while both CD36 and TREM2 are recognized for their roles in lipid uptake, emerging evidence suggests they also participate in regulation of cellular lipid processing and efflux.^23^ *Trem2*-deficient microglia have been reported to exhibit impaired clearance of myelin-derived cholesterol, which was associated with downregulation of genes involved in lipid catabolism and efflux, such as *Apoe* and *Abca1*, leading to accumulation of cholesteryl esters and lipid droplets^60^. Similarly, inhibition of CD36 has been shown to reduce activity of LXR and PPAR and promote inflammation in response to myelin treatment *in vitro*^23^. This is consistent with our data showing exacerbation of some inflammatory responses in *Cd36* but not *Trem2* deficient mice. We expect that in the absence of CD36 or TREM2, other receptors may partially compensate for lipid uptake, but not for the downstream regulation of lipid processing and efflux pathways.

Our imaging and lysosome-specific lipidomic data indicate that after TBI neutral lipids including cholesterol esters and triglycerides accumulate in both, lipid droplets and lysosomes. Additionally, we observed increase in lysosomal levels of ceramides and sphingomyelins. Cholesterol, ceramides and sphingomyelins are all major components of myelin, and their lysosomal accumulation supports myelin debris phagocytosis as a major source of lysosomal lipids after TBI. Triglycerides, however, are not enriched in myelin. Proteomic profiling revealed apolipoprotein E (APOE) as the top protein accumulating in lysosomes after TBI. As APOE is the major neutral lipid carrier in the brain,^61,62^ this suggests that triglycerides and potentially some cholesterols and may be derived from lipoprotein phagocytosis. Another protein enriched in TBI lysosomes was ANXA5, which has been shown to mediate phagocytic clearance of apoptotic cells and debris through direct interaction with phosphatidylserine on their membranes.^63^ In addition to its role as lipid carrier, APOE is well-established in modulation of lipid metabolism through the activation of LXR.^64^ Consistently, our scRNAseqidentified the LXR (NR1H3) pathway as the most significant regulatory network in the lipid-accumulating BAM_7 cluster. This convergence of multi-omics findings highlights the LXR pathway as a central regulator of lipid handling and inflammatory responses in monocytes following injury and suggests it as a compelling therapeutic target in TBI.

In peripheral phagocytes including macrophages uptake of certain lipids including cholesterol can directly cause lysosomal dysfunction. The proposed mechanisms include inhibition of vesicle fusion due to altered membrane composition, and physical disruption of lysosomal membranes.^65,66^ This has been documented in tissues high in cholesterol such as atherosclerotic plaques and demyelinated lesions in multiple sclerosis, where accumulation of lipid droplets in foam macrophages and lipid-loaded microglia is associated with lysosomal dysfunction, defective phagocytosis, and inflammation.^48^ Our data indicate that, similarly, phagocytosis of myelin debris after TBI affects lysosomal function and leads to inhibition of autophagy in affected microglia and monocytes. We recently demonstrated that TBI causes inhibition of microglial and monocyte autophagy and that this inhibition is an important contributing factor to excessive and prolonged neuroinflammatory responses.^16,18^ However, the mechanisms leading to autophagy inhibition after TBI remained unknown. The data presented here indicate that lipid and especially myelin phagocytosis induces lysosomal dysfunction and, consequently inhibition of autophagy in the injured brain. In addition to its role in maintenance of protein and organelle homeostasis and regulation of inflammation, recent data indicate that autophagy also regulates lipid metabolism.^19,21,22^ Autophagy has been shown to specifically target and degrade lipid droplets through lipophagy, thus promoting lipid efflux over storage. Consistently, we observed more pronounced lipid metabolism reprograming and exacerbated cellular lipid accumulation in mice with microglia/monocyte specific autophagy defects. Our data suggest a pathological feedback loop, where lipid phagocytosis causes inhibition of autophagy-lysosomal function after TBI, which in turn exacerbates cellular lipid retention, reprograming and inflammation.

Our study highlights lipid metabolism imbalance within mononuclear phagocyte populations as a central hallmark of the innate immune response in the context of TBI. This dysregulation manifests as lipid droplet accumulation, altered expression of genes involved in lipid processing, and a shift toward pro-inflammatory metabolic programs. Importantly, these changes are not restricted to TBI but appear to be a convergent feature across diverse forms of acute CNS inflammation. Similar lipid accumulation in microglia and/or macrophages has been documented in neuroinflammatory contexts such as spinal cord injury,^57^ ischemic stroke,^56^ and multiple sclerosis,^25^ suggesting a shared mechanism by which innate immune cells present in the CNS adapt their lipid metabolism in response to inflammation. These findings underscore the potential of targeting lipid metabolism pathways as a unifying therapeutic approach across multiple acute CNS pathologies where innate immune dysfunction is a key contributor. Additionally, lipid droplet associated microglia (LDAM) are observed and play a pathological role during brain aging and in neurodegenerative diseases.^1,9^ History of CNS injury including TBI is a strong predisposing factor to AD and other AD-related dementias later in life.^67–69^ Since the lipid accumulating microglial and monocyte populations we identified in the TBI brain strongly resemble LDAM, we expect that they may contribute to the accelerated development of dementia after injury.

## Supplementary Material

Supplementary Files

This is a list of supplementary files associated with this preprint. Click to download.

• MehrabaniTableS2.xlsx

• MehrabaniTableS4.xlsx

• MehrabaniTableS1.xlsx

• MehrabaniMaterialsandMethods.pdf

• MehrabaniTableS3.xlsx

• MehrabaniTableS6.xlsx

• MehrabaniTableS5.xlsx

• MehrabaniTableS7.xlsx

• NNA92663AMehrabaniSuppFiguresS1S6.pdf
